# Prognostic Value of Lymph Node Evaluation in Stage II Small Bowel Adenocarcinoma: An Updated Analysis of Surveillance, Epidemiology, and End Results Database

**DOI:** 10.3389/fonc.2022.865745

**Published:** 2022-03-25

**Authors:** Zhen Liu, Kun Liu, Jiale Gao, Chao Jing, Yanhong Ma, Shu Zheng, Jianzhen Shan

**Affiliations:** ^1^ Department of Oncology, The First Affiliated Hospital, Zhejiang University School of Medicine, Hangzhou, China; ^2^ Cancer Institute (Key Laboratory for Cancer Intervention and Prevention, China National Ministry of Education, Zhejiang Provincial Key Laboratory of Molecular Biology in Medical Sciences), The Second Affiliated Hospital, Zhejiang University School of Medicine, Hangzhou, China; ^3^ Department of General Surgery, Beijing Friendship Hospital, Capital Medical University, Beijing, China; ^4^ Clinical Center for Colorectal Cancer, Capital Medical University, Beijing, China; ^5^ Beijing Key Laboratory of Cancer Invasion and Metastasis Research, Beijing, China; ^6^ National Clinical Research Center for Digestive Diseases, Beijing, China

**Keywords:** small bowel adenocarcinoma, lymph node evaluation, cancer-specific survival, stage II patient, SEER database

## Abstract

**Background:**

Due to the lack of large-scale clinical trials, the treatment strategies of small bowel adenocarcinoma (SBA) are controversial, especially for stage II patients. According to the National Comprehensive Cancer Network (NCCN) guideline, few lymph nodes (LNs) examined (<5 for duodenum or <8 for jejunal/ileal primary location) are one of the high-risk features for stage II patients, for whom adjuvant chemotherapy is recommended. This consensus is originally drawn from data in the Surveillance, Epidemiology, and End Results Database (SEER) between 1988 and 2010. However, the surgical modalities and chemotherapy strategies changed a lot after 2004 for SBA patients. The previous data may not represent a true picture of current therapeutics. Thus, we reanalyzed the SEER database and updated the cutoff point of LN numbers resected with respect to cancer-specific survival (CSS) using the latest SEER information.

**Methods:**

Patients diagnosed with stage II SBA and who underwent curative surgery between 2004 and 2018 were extracted from the SEER database. CSS was calculated using the Kaplan–Meier method and compared by log-rank test. Maximum survival differences based on total LNs examined for duodenal and jejunoileal tumors were determined separately with the cut-point analysis and maximum log-rank χ^2^ statistic. A nomogram model was constructed based on the multivariate Cox analysis to predict 5‐ and 10‐year CSS and was then validated with an internal cohort.

**Results:**

A total of 935 stage II SBA patients met the inclusion criteria. The greatest difference in survival was found in patients who had removal of at least 5 LNs for duodenal and 12 LNs for jejunoileal tumors. Multivariate Cox analysis showed that age, T stage, histology grade, primary site, and LN numbers were independent prognostic factors for survival. The C index of nomogram model was 0.701 (95% CI, 0.661–0.741, p < 0.001).

**Conclusions:**

The number of LNs harvested is an important prognostic factor for survival in stage II SBA patients. LN number examined <5 remains a high-risk factor for duodenum, but the cutoff point for jejunal/ileal tumors should rise from 8 to 12. Appropriate radical lymphadenectomy should be performed in stage II SBA surgery.

## Introduction

Small bowel adenocarcinoma (SBA) is an uncommon cancer of the gastrointestinal tract, which accounts for less than 5% of all gastrointestinal malignancies (1). SBA is generally defined as duodenal, jejunal, and ileal adenocarcinoma. Its mechanisms are poorly understood (2). Due to the lack of large-scale, multicenter, randomized controlled trials, the optimal therapeutics are controversial. The clinical practices on SBAs usually follow the guidelines of colorectal cancers (CRCs), in which surgery is the main strategy for early-stage disease and chemotherapy for advanced disease (3).

Radical resection is the standard treatment for stage II SBAs. However, the efficacy of adjuvant treatment for these patients is unclear. Previous studies reported that the number of lymph nodes (LNs) removed during surgery is an essential indicator for evaluating the severity and survival of patients (4). The National Comprehensive Cancer Network (NCCN) guideline of SBA (Version 2.2021) suggested that inadequate LNs examined (<5 for duodenal or <8 for jejunal/ileal primary tumor location) was one of the high-risk features for stage II SBA patients, for whom adjuvant chemotherapy was recommended. This consensus was drawn from two studies conducted by Thuy et al. and Overman et al., which analyzed SBA patients from the Surveillance, Epidemiology, and End Results Database (SEER) (5, 6). The former study included patients from 1988 to 2010 and the latter from 1988 to 2005.

Nevertheless, the treatment strategies for intestinal cancers have changed a lot based on findings of several large clinical trials in the past 20 years, which remarkably prolonged the survival time of patients. Before the 2000s, 5-fluorouracil (5-FU) monotherapy was the standard adjuvant chemotherapy choice for CRC and SBA patients (7). Combined regimens of oxaliplatin and 5-FU have emerged as a new standard of care since 2002 (8, 9). Targeted anticancer agents such as bevacizumab and cetuximab have been approved by the Food and Drug Administration (FDA) since 2004 (10, 11). The advances of these new drugs improved clinical survival outcomes significantly.

Another fact is that surgical modalities for SBA and CRC improved a lot in the past 20 years. By analyzing data from the SEER database, we found that the median number of total LNs removed during surgery increased from 6 in 2004 to 15 in 2018 in stage II SBA patients.

Thus, we speculate that, since LNs as an essential prognostic factor, the data before the 2000s may not exactly represent the current strategies for SBA treatment, especially for patients with stage II disease. In this study, we updated the LN number as a risk factor for stage II SBA patients with the latest data from the SEER database between 2004 and 2018 and indeed found that the cutoff value of LNs removed for jejunum and ileum should be changed.

## Materials and Methods

### Patients

All patients diagnosed as SBA (ICD-O-3:8140, 8143, 8144, 8145, 8210, 8211, 8220, 8255, 8260, 8261, 8262, 8263, 8310, 8480, 8481, and 8490) were extracted from the SEER database (http://seer.cancer.gov/) with SEER*Stat Software (version 8.3.9.2). To ensure comparable treatment methods with current clinical practice, only patients diagnosed after 2004 were included. Parameters included age, sex, tumor size, primary tumor site, LNs examined, distant metastasis, histological grade, surgery, survival time, and survival status. Tumor size and extension (T), LN metastases (N), and distance metastases status (M) were combined to classify patients to stage I to IV according to the American Joint Committee on Cancer (AJCC) 8th edition cancer staging system. Stage II patients with complete clinical information who underwent surgery and SBA as the first diagnosed tumor were included for further analysis.

### Cut-Point Analysis for Total Lymph Nodes

Cancer-specific survival (CSS) was calculated using the Kaplan–Meier method, and comparisons were analyzed by the log-rank test. Patients were censored if they died from causes other than SBA or if they were alive at follow-up. Maximum survival differences based on total LNs examined were determined separately with the cut-point analysis and maximum log-rank χ^2^ statistic (12). The point with maximum likelihood ratio chi-square value was selected as the optimal cut-point. The number of LNs removed lower than the cut-point value was regarded as high risk, while the LNs equal or higher than the cut-point value were marked as low risk.

### Nomogram Construction

Patients were randomly assigned to a training cohort (70%) and a validation cohort (30%).

Univariate correlations between prognostic variables and CSS were carried out using the Kaplan–Meier method and log-rank test. Factors with a significant difference from univariate analysis were further analyzed in a multivariate Cox proportional hazards model. A nomogram model was constructed based on the training cohort to predict the 5‐ and 10‐year CSS with the rms package in R software version 3.5.1. The nomogram was then validated on the validation cohort. The discriminative capacity of the nomogram was evaluated with the concordance index (C-index). Calibration plots were constructed to compare the calibrations between nomogram-predicted and actual survival.

### Statistical Analysis

Means and SDs for continuous variables were determined using descriptive statistics. Categorical and discrete variables were compared with the χ^2^ test. Continuous variables were expressed as median with interquartile range. Student’s t-test was used to compare means. Multivariate analyses were performed by multivariate Cox proportional hazards regression model using IBM SPSS (vision 26.0). Nomogram was generated by R software with the “rms” package (www.r-project.org). Hazard ratios (HRs) and their 95% CI were calculated. A two-sided p-value <0.05 was considered statistically significant.

### Ethics Statement

This study was exempted from Institutional Review Board review because the SEER database contained no personal identifiers.

## Results

### Patient Characteristics

**Figure 1:**
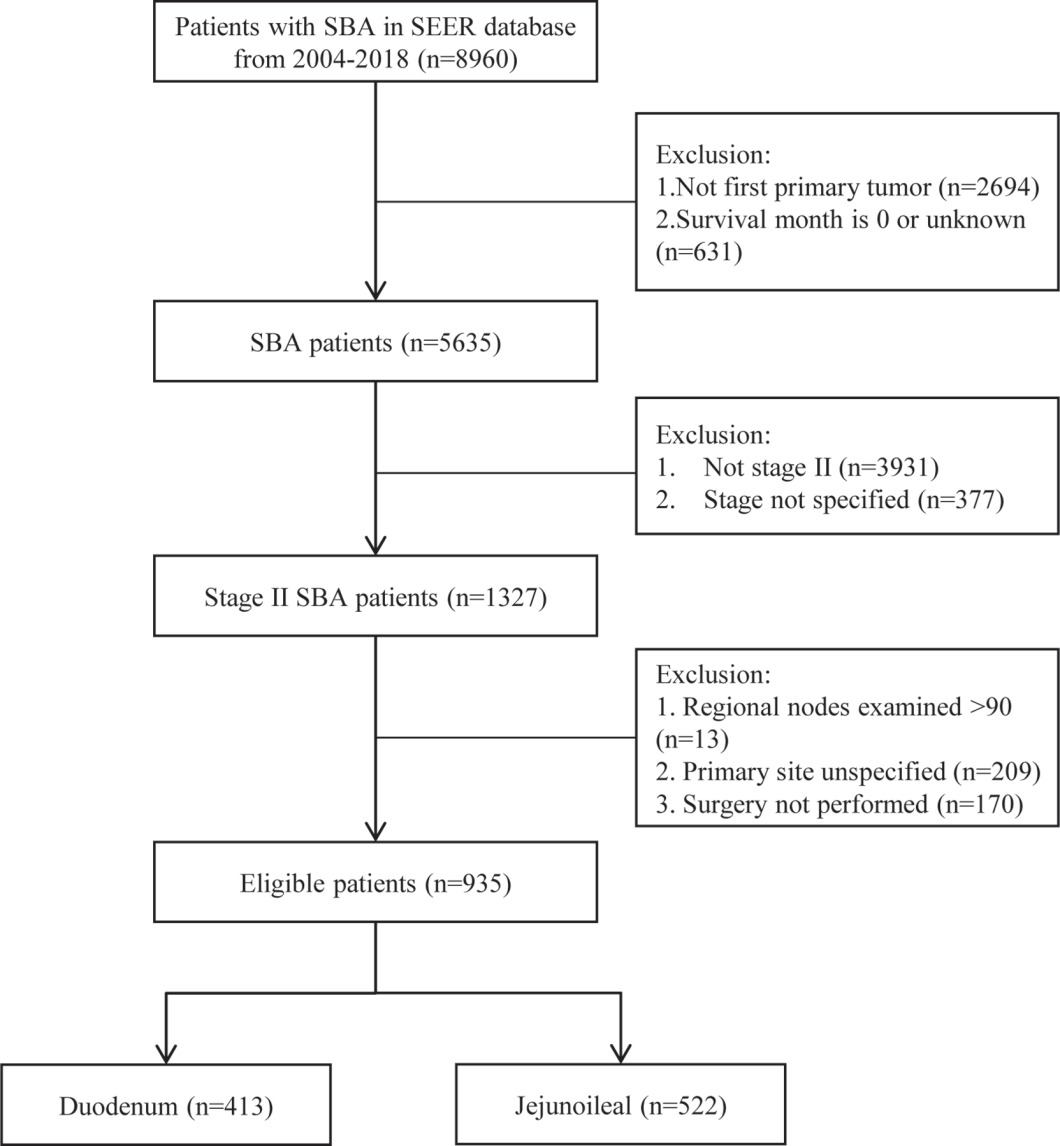
Flowchart demonstrating the selection processes of cases from SEER database used in this analysis. SBA, small bowel adenocarcinoma; SEER, Surveillance, Epidemiology, and End Results Database.

From 2004 to 2018, 8,960 adults were diagnosed with SBA. A total of 2,694 cases were excluded because it was not their first diagnosed tumor, and 631 cases were excluded due to missing survival time (survival month was 0 or unknown). Out of 5,635 primary SBA patients, 1,327 patients were under stage II. The numbers of LN resected were unknown among 13 patients. The primary site of 209 patients was unspecified, and 170 patients did not undergo surgery. Finally, a total of 935 SBA patients with stage II and underwent surgery were enrolled, including 413 (44.2%) in the duodenum and 522 (55.8%) in the jejunoileum (**Figure 1**).

As shown in **Table 1**, two patient cohorts were comparable with respect to gender, age, grade, ethnicity, and area. However, the median number of LNs resected was prone to be higher in the duodenum than jejunoileal cohort, which was 11 (95% CI, 10–12) and 9 (95% CI, 8–11), respectively. There were more patients with the T4 stage in the duodenum (43.6%) than the jejunoileum (36.6%).

**Table 1:** Clinicopathological characteristics of patients with small bowel adenocarcinoma.

	Entire cohort, n = 935	Duodenum, n = 413	Jejunoileum, n = 522
Gender (%)			
Male	482 (51.6)	211 (51.1)	271 (51.9)
Female	453 (48.4)	202 (48.9)	251 (48.1)
Age (%)			
<45	94 (10.1)	37 (9.0)	57 (10.9)
45–64	394 (42.1)	164 (39.7)	230 (44.1)
≥65	447 (47.8)	212 (51.3)	235 (45.0)
T stage (%)			
T3	564 (60.3)	233 (56.4)	331 (63.4)
T4	371 (39.7)	180 (43.6)	191 (36.6)
Median number of LNs examined (95% CI)	10 (9-11)	11 (10-12)	9 (8-11)
Histology grade (%)			
Well	94 (10.1)	34 (8.2)	60 (11.5)
Moderate	490 (52.4)	206 (50.0)	284 (54.4)
Poor	250 (26.7)	120 (29.1)	130 (24.9)
Undifferentiated	15 (1.6)	7 (1.7)	8 (1.5)
Unknown	86 (9.2)	46 (11.1)	40 (7.7)
Year diagnosed (%)			
2004–2008	281 (30.1)	132 (32.0)	149 (28.5)
2009–2013	310 (33.2)	137 (33.2)	173 (33.1)
2014–2018	344 (36.8)	144 (34.9)	200 (38.3)
Ethnicity (%)			
White	712 (76.1)	324 (78.5)	388 (74.3)
Black	167 (17.9)	63 (15.3)	104 (19.9)
Asian	43 (4.6)	22 (5.3)	21 (4.0)
Others	13 (1.4)	4 (0.9)	9 (1.7)
Area (%)			
Urban	838 (89.6)	375 (90.18)	463 (88.7)
Rural	97 (10.4)	38 (9.2)	59 (11.3)

LNs, lymph nodes.

### Cut-Point Analysis for Total Lymph Nodes

To identify the optimum cut-point number of LNs that should be resected during surgery, maximum log-rank χ^2^ analysis was performed. We found that the greatest difference in survival was found in patients who had removals of at least 5 LNs for duodenal and 12 LNs for jejunoileal tumors (**Table 2**).

**Table 2:** Cut-point analyses for detecting optimal number of lymph nodes with respect to cancer-specific survival.

No. of LNs examined	Duodenum		Jejunoileum	
	χ^2^	p-Value	χ^2^	p-Value
1 vs. ≥2	12.95	0.0003	1.17	0.2795
≤2 vs. ≥3	11.73	0.0006	1.973	0.1602
≤3 vs. ≥4	9.54	0.002	2.281	0.131
≤4 vs. ≥5	**20.69**	**<0.0001**	1.823	0.1769
≤5 vs. ≥6	19.76	<0.0001	1.184	0.2766
≤6 vs. ≥7	17.84	<0.0001	1.268	0.2602
≤7 vs. ≥8	10.76	0.001	1.968	0.1606
≤8 vs. ≥9	14.03	0.0002	3.478	0.0622
≤9 vs. ≥10	11.72	0.0006	3.476	0.0623
≤10 vs. ≥11	10.06	0.0015	3.486	0.0619
≤11 vs. ≥12	15.65	<0.0001	**4.253**	**0.0392**
≤12 vs. ≥13	13.95	0.0002	3.168	0.0751
≤13 vs. ≥14	13.72	0.0002	3.672	0.0553
≤14 vs. ≥15	13.15	0.0003	3.418	0.0645
≤15 vs. ≥16	13.83	0.0002	3.713	0.054
≤16 vs. ≥17	14.2	0.0002	4.061	0.0439
≤17 vs. ≥18	12.72	0.0004	3.244	0.0717

Values in bold indicate the highest χ^2^ and statistical significance.

LNs, lymph nodes.

**Figure 2:**
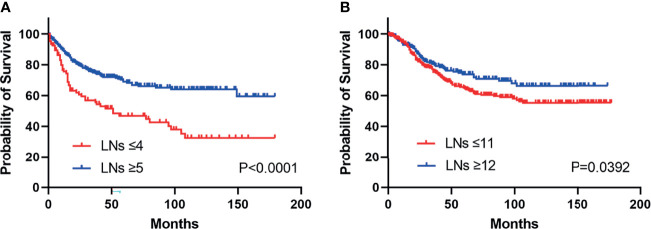
Kaplan–Meier curves illustrate cancer-specific survival stratified by lymph node risk in patients with duodenal (HR: 0.39, 95% CI, 0.26 to 0.58, p < 0.0001) **(A)** and jejunoileal tumors (HR: 0.71, 95% CI, 0.52 to 0.98, p = 0.0392) **(B)**. LNs, lymph nodes.

Kaplan–Meier survival curves are shown in **Figure 2**. Patients above the cut-off point showed greater benefits in median survival for both duodenal (HR: 0.39, 95% CI, 0.26 to 0.58, Pearson’s chi-square: 20.69, p < 0.0001) and jejunoileal tumors (HR: 0.71, 95% CI, 0.52 to 0.98, Pearson’s chi-square: 4.253, p = 0.0392).

### Multivariable Survival Analysis

Many factors were found to influence the CSS. The multivariate Cox regression was performed to explore potential factors that might be associated with the survival time. As shown in **Table 3**, age, T stage, histology grade, primary site, and LNs examined were independent prognostic factors for survival.

**Table 3:** Multivariate Cox regression analyses of patients with small bowel carcinoma.

	HR	95% CI	p-Value
Age at diagnosis			
<45	Reference		
45–64	1.069	0.679–1.683	0.772
≥65	1.787	1.152–2.773	0.009
T stage			<0.001
T3	Reference		
T4	2.076	1.635–2.636	<0.001
Histology grade			
Well	Reference		
Moderate	1.412	0.884–2.256	0.148
Poor	1.827	1.125–2.967	0.014
Undifferentiated	2.425	1.060–5.550	0.035
Unknown	1.297	0.631–2.670	0.479
Primary site			
Duodenum	Reference		
Jejunoileum	0.744	0.565–0.981	0.036
LNs examined			
High risk	Reference		
Low risk	0.521	0.401–0.678	<0.001

HR, hazard ratio; LNs, lymph nodes.

We can see that older age (≥45 years), T4, and higher histology grade were negative prognostic factors. Tumors located in the jejunoileum had a better CSS than in the duodenum (HR: 0.745, 95% CI, 0.565–0.981, p < 0.05). LN risks based on the cut-point analysis (5 for duodenum and 12 for jejunoileum) were significantly related to CSS (low risk/high risk, HR: 0.522, 95% CI, 0.401–0.678, p < 0.001).

### Nomogram Construction and Validation

The multivariate Cox regression model revealed that CSS was associated with age, T stage, pathological grade, tumor location, and LNs risks. Based on these variables, we established a CSS nomogram to predict the 5- and 10-year survival rates of SBA patients.

**Figure 3:**
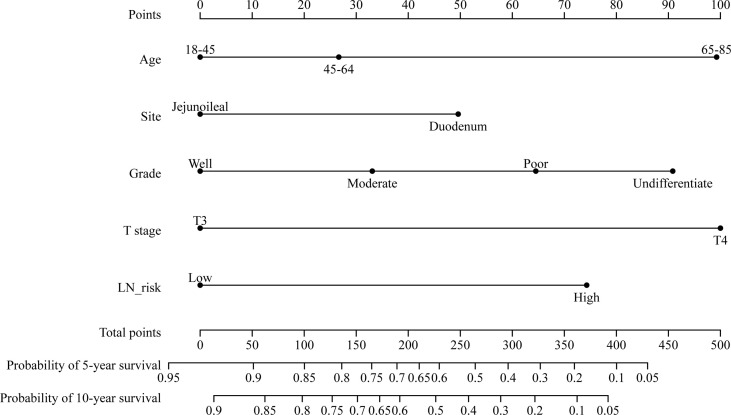
Nomogram predicting the 5- and 10-year cancer-specific survival of patients with stage II small bowel adenocarcinoma. LN risk, lymph node risk.

The nomogram in **Figure 3** shows that pathological grade at diagnosis was the greatest contributor to the prognosis, followed by T stage, LN risk, age, and primary site. The C index of this model was 0.701 (95% CI, 0.661–0.741, p < 0.001).

**Figure 4:**
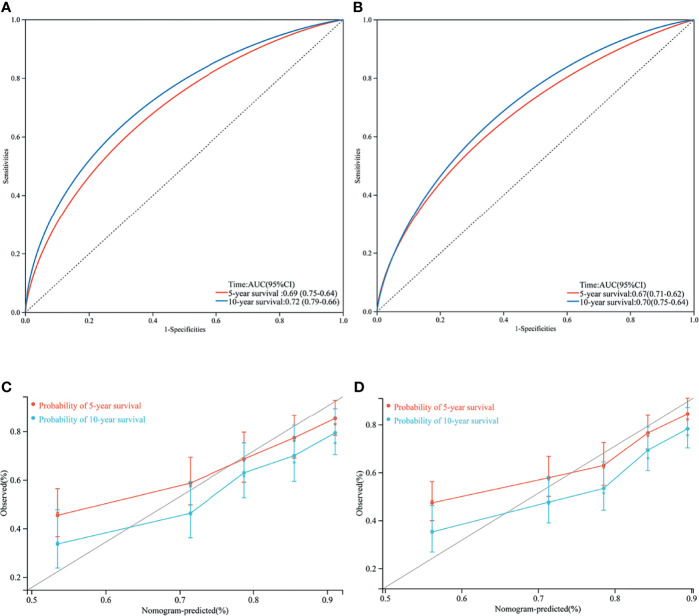
Area under curve (AUC) of the training set **(A)** and validation set **(B)**; 5- and 10- year cancer-specific survival according to the training set **(C)** and the validation set **(D)**.

We performed an internal validation of this nomogram. The results showed that the C-index of the validation nomogram was 0.687 (95% CI 0.653–0.721, p < 0.001). The area under the curve (AUC) of 10-year survival was 0.72 (95% CI 0.66–0.79) for the training cohort and 0.70 (0.64–0.75) for the validation cohort (**Figures 4A, B**).

Calibration plots revealed a strong correlation between predictions estimated by the nomogram and actual observations for both the training and validation cohorts, especially for the 10-year CSS (**Figures 4C, D**).

## Discussion

SBA treatment is challenging, given its relative rarity and advanced stage of diagnosis. Due to the lack of large-scale, multicenter, randomized, controlled trials, the treatment strategies usually follow the consent of patients with CRCs. It is well accepted that patients with stage II CRC should be divided into a high-risk group and low-risk group according to several postoperative histology features, such as T4, grade 3 or 4, lymphovascular or perineural invasion, and <12 LNs examined (13). Patients with these high-risk features should receive adjuvant chemotherapy after the initial surgery (14). Similar evaluations had also been made for SBA. High-risk features for stage II SBA patients include T4 stage, close or positive surgical margins, few LNs examined (<5 for duodenal or <8 for jejunal/ileal primary tumor location), or tumor perforation according to NCCN guidelines. Our study focused on one of these features, LNs examined, and found that the optimal cut-off numbers for stage II SBA patients should be updated to 12 for jejunal/ileal primary tumor location, which was consistent with stage II CRC patients.

**Figure 5:**
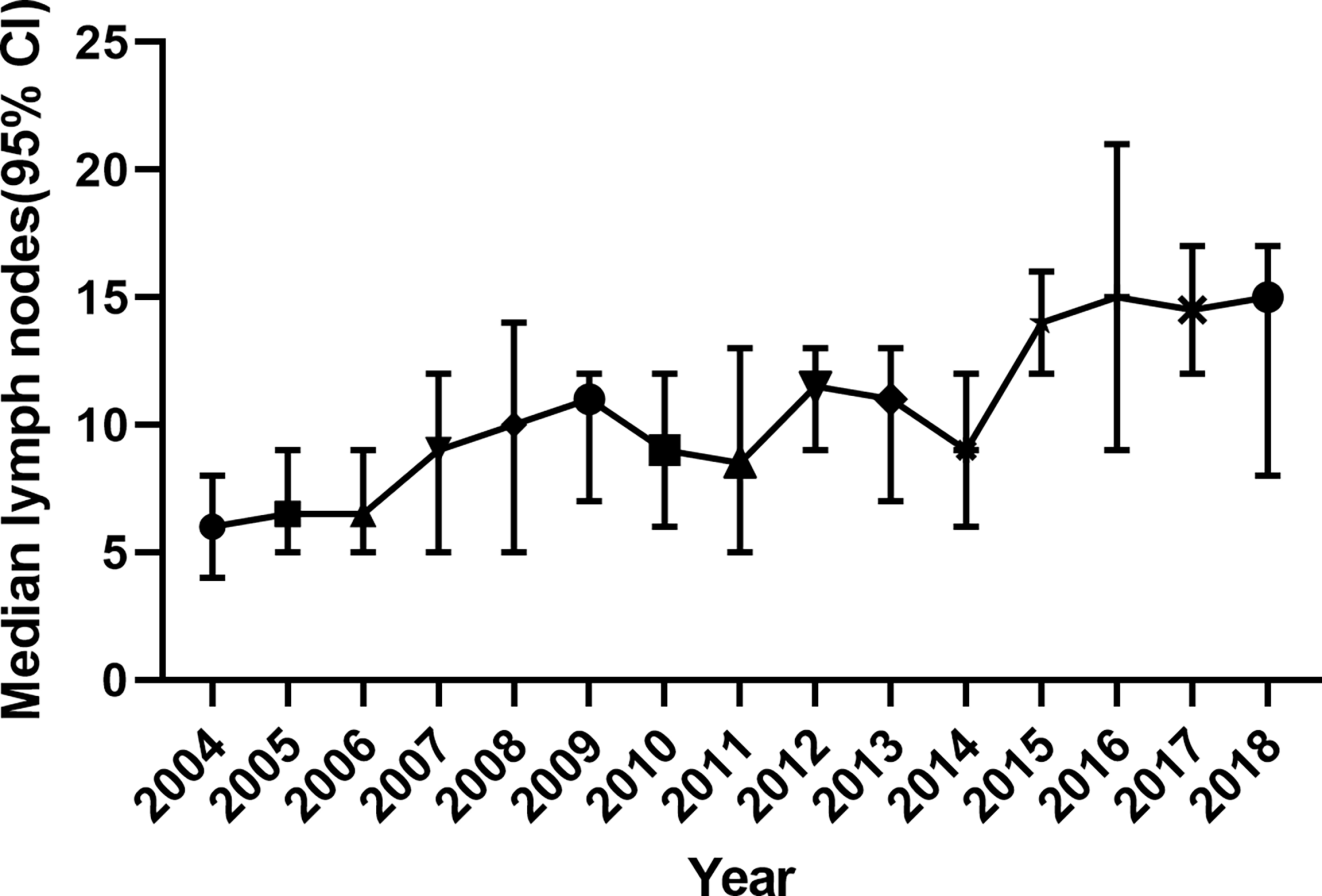
The number of surgically removed lymph nodes in stage II SBA patients from 2004 to 2018, indicated by median and 95% CI. SBA, small bowel adenocarcinoma.

The recommendation of LN risks in the NCCN guideline (<5 for duodenal or <8 for jejunal/ileal primary tumor location) is drawn from patients in the SEER database from 1988 to 2010 and 1988 to 2005 (5, 6). However, the treatment strategies changed significantly in recent years. A recent study demonstrated that the LN numbers in NCCN guidelines (<5 for duodenal or <8 for jejunal/ileal primary tumor location) did not discriminate SBA patients from high and low risk (p = 0.166) (15). In **Figure 5**, we can see that the median number of surgical removed LNs in stage II SBA patients is increasing, from 6 (95% CI, 6–8) in 2004 to 15 (95% CI, 8–17) in 2018. On the other hand, the addition of oxaliplatin to fluorouracil-based chemotherapy has become the standard treatment since 2002, which is more efficient than 5-FU monotherapy (9). Targeted anticancer agents such as bevacizumab and cetuximab have been developed since 2004. These strategies improved survival time significantly. Thus, the outcomes of SBA patients before 2004 may not represent a true picture of current treatments.

Our study updated the survival data using the latest SEER database in patients with stage II SBA diagnosed between 2004 and 2018. We demonstrated that patients with stage II SBA and total examined LNs <5 for duodenal tumors or <12 for jejunal/ileal primary tumors were at high risk. These patients may benefit from adjuvant chemotherapy. We also established a nomogram to predict the 5- and 10-year survival rates of SBA patients, which was more accurate than the AJCC staging system.

Several limitations exist in our analysis. There is a lack of treatment information in the SEER database, such as surgical margins, tumor perforation, chemotherapy regimen, recurrence, and molecular features. We cannot compare the survival outcome directly between patients with and without adjuvant chemotherapy. SEER database is retrospective, and we excluded patients with incomplete information, which may lead to selection bias. Though 935 patients were finally included in our study, large multicenter, randomized clinical trials are strongly needed to further confirm the conclusion. The BALLAD trial (NCT02502370) is ongoing, which is the first prospective trial designed to recruit 100 participants and to assess the benefit of adjuvant chemotherapy versus observation alone in patients with stage I–III SBA. Primary outcomes are 3-year disease-free survival and overall survival (OS), with expected results in 2023.

## Conclusion

The total number of LNs harvested is an important prognostic factor of survival in stage II SBA. Patients with total examined LNs <5 for duodenal or <12 for jejunal/ileal primary tumor are at high risk. Sufficient LN dissection is recommended for stage II SBA patients.

## Data Availability Statement

The original contributions presented in the study are included in the article/supplementary material. Further inquiries can be directed to the corresponding authors.

## Ethics Statement

Ethical review and approval were not required for the study on human participants in accordance with the local legislation and institutional requirements. Written informed consent for participation was not required for this study in accordance with the national legislation and the institutional requirements.

## Author Contributions

ZL, SZ, and JS designed the study. ZL, KL, JG, CJ, and YM collected and analyzed the data. ZL and KL organized the manuscript. All authors have read and approved the final manuscript.

## Funding

This work is supported by grants from the National Natural Science Foundation of China (81602635), Zhejiang Provincial Natural Science Foundation (LY21H160042), and Zhejiang Medical and Health Project (2017205840, 2018274816, 2019334185).

## Conflict of Interest

The authors declare that the research was conducted in the absence of any commercial or financial relationships that could be construed as a potential conflict of interest.

## Publisher’s Note

All claims expressed in this article are solely those of the authors and do not necessarily represent those of their affiliated organizations, or those of the publisher, the editors and the reviewers. Any product that may be evaluated in this article, or claim that may be made by its manufacturer, is not guaranteed or endorsed by the publisher.
